# Genetic connectivity from the Arctic to the Antarctic: *Sclerolinum contortum* and *Nicomache lokii* (Annelida) are both widespread in reducing environments

**DOI:** 10.1038/s41598-018-23076-0

**Published:** 2018-03-19

**Authors:** Mari H. Eilertsen, Magdalena N. Georgieva, Jon A. Kongsrud, Katrin Linse, Helena Wiklund, Adrian G. Glover, Hans T. Rapp

**Affiliations:** 1 Department of Biological Sciences, University of Bergen, PO Box 7800, N-5020 Bergen, Norway; 2 K.G. Jebsen Centre for Deep-Sea Research, University of Bergen, PO Box 7803, N-5020 Bergen, Norway; 3 Life Sciences Department, Natural History Museum, Cromwell Road, London, SW7 5BD UK; 4 Department of Natural History, University Museum of Bergen, PO Box 7800, N-5020 Bergen, Norway; 5 British Antarctic Survey, High Cross, Madingley Road, Cambridge, CB3 0ET UK; 6 Uni Research, Uni Environment, PO Box 7810, N-5020 Bergen, Norway

## Abstract

The paradigm of large geographic ranges in the deep sea has been challenged by genetic studies, which often reveal putatively widespread species to be several taxa with more restricted ranges. Recently, a phylogeographic study revealed that the tubeworm *Sclerolinum contortum* (Siboglinidae) inhabits vents and seeps from the Arctic to the Antarctic. Here, we further test the conspecificity of the same populations of *S. contortum* with additional mitochondrial and nuclear markers. We also investigate the genetic connectivity of another species with putatively the same wide geographic range - *Nicomache lokii* (Maldanidae). Our results support the present range of *S. contortum*, and the range of *N. lokii* is extended from vents and seeps in the Nordic Seas to mud volcanoes in the Barbados Trench and Antarctic vents. *Sclerolinum contortum* shows more pronounced geographic structure than *N. lokii*, but whether this is due to different dispersal capacities or reflects the geographic isolation of the sampled localities is unclear. Two distinct mitochondrial lineages of *N. lokii* are present in the Antarctic, which may result from two independent colonization events. The environmental conditions inhabited by the two species and implications for their distinct habitat preference is discussed.

## Introduction

The geographic ranges of marine species are determined by a number of factors, and among the most important are the spatial distribution of suitable habitat and the dispersal potential of the species. The traditional view has been that biogeographic ranges in the deep sea are wide, due to a homogenous environment and an apparent lack of barriers to dispersal^1^. This paradigm has been challenged by molecular studies, which have revealed that many deep-sea morphospecies with wide geographic ranges are in fact several species confounded under the same name^2–5^, casting doubt on the prevalence of widespread species in the deep sea. In contrast to the continuous and apparently uniform sedimented habitats of the abyssal plains, deep-sea reducing environments (hydrothermal vents, cold seeps and organic falls), have a discontinuous and patchy distribution. Hydrothermal vents are found in tectonically active areas such as mid-ocean ridges, cold seeps are found along continental margins, and organic falls are usually found close to the continents or along cetacean migration routes^6^. Reducing environments host ecosystems that are based on chemosynthetic primary production (chemosynthesis-based ecosystems; CBEs), and a high proportion of the species found in CBEs are dependent on a chemosynthesis-based food source, and are thus restricted to these habitats^7^.

The fragmented distribution of reducing environments implies that CBE-restricted species have to disperse across areas of unsuitable habitat to colonize new localities and to maintain connectivity between populations. Apart from some taxa with highly mobile adults, such as shrimps, invertebrates mainly disperse in the larval stage, and the dispersal potential is therefore related to characteristics such as egg size and larval developmental mode^8^. Species with lower dispersal potential will be more affected by gaps in the distribution of suitable habitats, and this can be seen by higher degrees of genetic structure or even vicariant speciation across habitat discontinuities^8,9^. Reproductive characteristics that are associated with good dispersal capabilities and low levels of genetic structure over long distances in CBE-adapted invertebrates are planktotrophic larvae (e.g. *Bathymodiolus* bivalves^10,11^ and *Alvinocaris*/*Rimicaris* shrimps^12,13^), arrested larval development (e.g. *Alvinella pompejana*^14^) or lecithotrophic larvae with large yolk reserves (e.g. *Branchipolynoe* spp.^15^). The level of habitat specificity may also affect the biogeographic ranges of CBE-adapted species. Although present data indicates that most CBE-adapted species are specific to either vents, seeps or organic falls^16,17^, there are some species that are able to inhabit multiple types of CBEs^4,13,18^. CBE generalists will have a higher number of suitable habitat patches available, which may enable them to maintain genetic connectivity over a larger geographical area. However, there are few studies of genetic connectivity between different CBEs^13,18^, and the importance of various CBEs as stepping stones for dispersal is debated^19,20^.

Biogeographic studies of hydrothermal vent fauna have revealed 5 distinct biogeographic provinces: the Northeast Pacific, East Pacific Rise, Western Pacific, Indian Ocean and Mid-Atlantic Ridge, with 95% of species endemic to one province^21^. CBEs in high latitudes (Arctic and Antarctic) have been less studied due to their remoteness and challenging weather conditions, but the presence of unique fauna indicates that these areas might comprise another two provinces^22,23^. The low proportion of shared species between provinces would indicate that most CBE-adapted species lack the ability to disperse over very long ranges. However, the degree of endemism may be overestimated due to taxonomic uncertainties and lack of comprehensive sampling^13^, and there is a need for integrative taxonomic work to evaluate the geographic ranges of CBE-adapted species^24^.

The siboglinid worm *Sclerolinum contortum* has the widest known range of CBE-adapted species, spanning nearly 16 000 km from the Arctic to the Antarctic^25^. *Sclerolinum contortum* was first described from a cold seep habitat at the Håkon Mosby mud volcano (HMMV) in the Arctic^26^, but has also been found at the sedimented hydrothermal vents at Loki’s Castle on the Arctic Mid-Ocean Ridge^23^, at cold seeps in the Gulf of Mexico (GoM)^27^ and at sedimented hydrothermal vents in the Bransfield Strait in the Antarctic^25^. Although there is some morphological variation between the populations^25,27^, their conspecificity was supported by a multigene phylogenetic analysis and population genetic analysis of the mitochondrial marker COI^25^. At Loki’s Castle, the tubes of *S. contortum* generate a mat-like structure together with the maldanid worm *Nicomache lokii* and other tube dwelling annelids in the diffuse-flow area of the vent field^28–30^. While *S. contortum* lacks an alimentary tract, and relies on energy from sulphide-oxidizing bacterial symbionts^31,32^, *N. lokii* is a grazer^28^. Stable isotope data indicates that *N. lokii* feeds on bacterial mats, mixed with particles sinking down from the pelagic zone^28^.

Here we present new findings of worms morphologically identified as *N. lokii* from the HMMV, the Barbados Trench mud volcanoes^33^ and from hydrothermal vents in the Antarctic. If these populations are in fact conspecific, it would extend the range of *N. lokii* from the Arctic to the Barbados Trench and the Antarctic, giving it a similar range as *S. contortum*. It would also expand the habitats occupied by *N. lokii* to include cold seeps. This study aims to: 1) further test the conspecificity of the populations of *S. contortum* from the Arctic, Gulf of Mexico and Antarctic with additional mitochondrial and nuclear markers, 2) test the conspecificity of the populations of *N. lokii* from the Arctic, Barbados Trench and the Antarctic, 3) compare the genetic divergence and putative population connectivity from the Arctic to the Antarctic of *S. contortum* and *N. lokii* and 4) assess how the environmental conditions inhabited by these two species may relate to their habitat preference and distributions.

## Materials and Methods

Specimens of *Nicomache lokii* were collected from the diffuse venting area at Loki’s Castle and from the Håkon Mosby Mud Volcano during University of Bergen cruises with the RV G.O. Sars in the period 2008–2016, from the Barbados Trench mud volcanoes during the SeepC Barbados cruise in 2012 (samples provided by CL Van Dover), and from hydrothermal vents in the East Scotia Sea during two cruises with the vessel RRS *James Cook*; JC42 in January–February 2010 and JC80 in December 2012. Collection details are listed in Table 1. A map of the sampling localities was generated using QGIS^34^.

**Table 1:** Sampling sites for *Nicomache lokii*.

	Site	Latitude	Longitude	Depth (m)
Arctic	Lokis Castle	73,5662	8,1585	2357
	HMMV	71,9975	14,7329	1262
Barbados	Atalante East	13,8282	−57,6447	4930
	Manon	13,7777	−57,5426	4742
Antarctic	E2 JC42	−56,0800	−30,3100	2608
	E2 JC80	−56,0883	−30,3182	2646
	E2 Cindy’s Castle	−56,0883	−30,3187	2646
	E2 Crab City	−56,0883	−30,3180	2641
	E2 Sepia JC80	−56,0883	−30,3182	2619
	Kemp Caldera Clam Road	−59,4100	−28,2100	1400
	E9 JC42	−60,0200	−29,5800	2400

For *Sclerolinum contortum*, the same DNA extractions were used as in Georgieva *et al*.^25^, and sampling and DNA extraction of *S. contortum* is described in that paper. DNA of *Nicomache lokii* was extracted using the QIAGEN DNeasy Blood and Tissue Kit, following the manufacturer’s protocol (spin-column protocol).

### PCR and sequencing

Four markers were selected for population genetic analyses: the mitochondrial Cytochrome C oxidase subunit I (COI), 16S rRNA and Cytochrome B (CytB), and the nuclear 28S rRNA (for primers see Supplementary Table S1). COI had already been sequenced for the three populations of *S. contortum* for a previous paper^25^, but the other three markers were sequenced for this study. The same four markers were used for species delimitation analyses of both *N. lokii* and *S. contortum*. For phylogenetic analysis of *Nicomache*, we used three of the population level markers, COI, 16S and 28S, and also the more conservative nuclear marker 18S rRNA. Three additional species of *Nicomache* were sequenced (*N. lumbricalis*, *N. minor* and *N. quadrispinata*), and also one species of the closely related genus *Petaloproctus* (*Petaloproctus tenuis*) as outgroups. All sequences used for the phylogenetic analyses, including representative sequences of all haplotypes were submitted to GenBank, see Supplementary Tables S2–S4 for accession numbers. PCR reactions and cycling profiles are listed in Supplementary methods.

Quality and quantity of amplicons were assessed by gel electrophoresis imaging using a FastRuler DNA Ladder (Life Technologies) and GeneSnap and GeneTools (SynGene) for image capture and band quantification. When multiple bands were present, the total PCR product was run on a new gel and the target band was extracted from the gel using MinElute Gel Extraction Kit (QIAGEN) following the manufacturer’s protocol. In cases where there were multiple weak bands, a small piece of the desired band was cut out from the gel and re-amplified using the original PCR protocol. Successful PCRs were purified using Exonuclease 1 (EXO, 10 U mL^−1^) and Shrimp 90 Alkaline Phosphatase (SAP, 10 U mL^−1^, USB Europe, Germany) in 10 μL reactions (0.1 mL EXO, 1 μL SAP, 0.9 μL ddH 2 O, and 8 μL PCR product). Samples were incubated at 37 °C for 15 min followed by an inactivation step at 80 °C for 15 min. The purified PCR products were sequenced using BigDye v3.1 (Life Technologies) and run on an Automatic Sequencer 3730XL at the sequencing facility of the Institute of Molecular Biology, University of Bergen. COI, 16S and 18S were sequenced with forward and reverse primers, while CytB and 28S were mainly sequenced with forward primers only.

### Sequence assembly and alignments

Forward and reverse sequences were assembled in Geneious v.6.1.8 (Biomatters Ltd.) and checked for contamination using BLAST^35^. For the protein coding genes (COI and CytB), sequences were translated to amino acids in Geneious to check for stop codons. Sequences were aligned in Geneious using MUSCLE^36^, except the 16S dataset for phylogenetic analysis of *N. lokii* which was aligned with MAFFT v7 using the online server^37^. Alignments for population genetic analyses were trimmed to the shortest sequence, while for the phylogenetic analysis missing data at the ends were coded with question marks.

### Population genetic analyses

**Figure 1:**
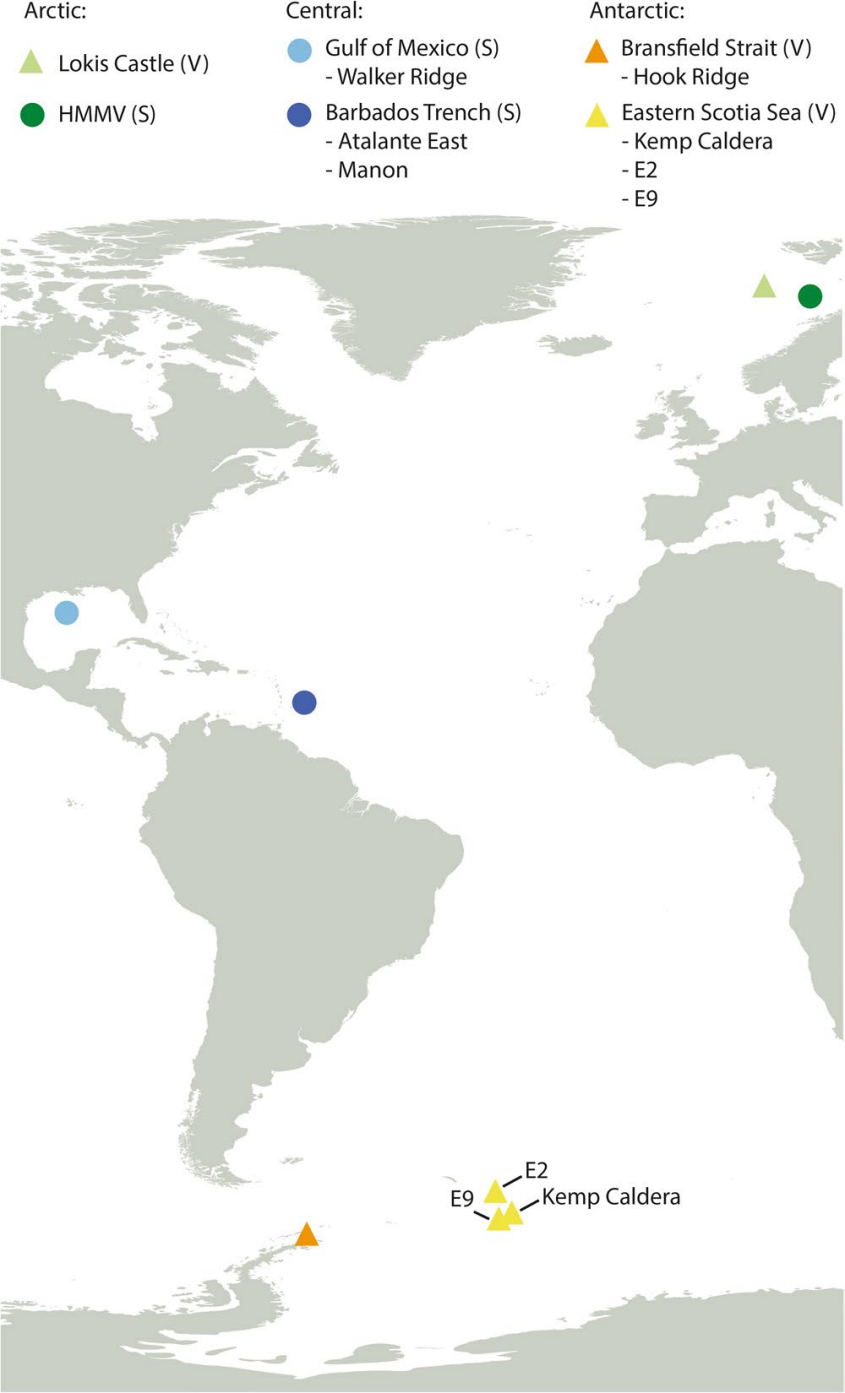
Map of sampling stations for *Sclerolinum contortum* and *Nicomache lokii* generated in QGIS^34^. The two stations in the Barbados Trench were located so close together that these are indicated with only one circle. There were also multiple sampling stations on the E2 ridge in the eastern Scotia Sea (see Table 1).

To obtain a good number of samples per population, samples from multiple sampling stations in the Barbados Trench and eastern Scotia Sea were pooled for population genetic analyses (see Fig. 1 and Table 1). The two sampling stations in the Barbados Trench (Atalante East and Manon) are closely located, while the Scotia Sea stations are further apart (approximately 400 km between E2 and E9, see Table 1 for coordinates). However, the same haplotypes were mainly found in all the Scotia Sea stations (see Results). Since HMMV is a cold seep, samples from this locality were indicated with a darker shade of green in the haplotype networks to separate it from the samples from the Loki’s Castle vent field. However, the HMMV and Loki’s Castle samples did not show much genetic variation, if any, in the haplotype networks, and these samples were pooled for the subsequent analyses. Between 10–35 specimens were sequenced from each population for each of the four markers (see Table 2). Six heterozygous individuals were identified for 28S by double peaks in the chromatograms, and out of these two were heterozygous for more than one position (Antarctic haplotypes A and B). Although the inference of haplotypes in individuals with multiple heterozygous positions is not straightforward, in this case the heterozygous positions matched the polymorphic positions between the only two haplotypes present in the population (as seen in homozygotes), and it was assumed that the individuals carried one copy of each of these haplotypes. For homozygotes, the sequences were duplicated to generate a dataset with two sequences per individual.

**Table 2:** Genetic diversity within populations.

	*Nicomache lokii*									*Sclerolinum contortum*								
		n	k	H ± SD	π ± SD	P	S/N	In-Dels	M (%)		n	k	H ± SD	π ± SD	P	S/N	In-Dels	M (%)
COI (656/547 bp)	**Arc**	23	5	0.597 ± 0.098	0.00178 ± 0.00032	4	4/0	0	0.18	**Arc**	23	6	0.700 ± 0.088	0.002 ± 0.0004	5	4/1	0	0.22
	**Bar**	35	5	0.64 ± 0.066	0.00894 ± 0.00061	14	14/0	0	0.91	**GoM**	15	7	0.781 ± 0.102	0.004 ± 0.0008	10	10/0	0	0.37
	**Ant**	32	2	0.444 ± 0.064	0.01488 ± 0.00216	22	22/0	0	1.54	**Ant**	27	1	0	0	0	0	0	0
	**Tot**	90	12	0.852 ± 0.017	0.01803 ± 0.00113	33	33/0	0	1.86	**Tot**	65	14	0.784 ± 0.042	0.00736 ± 0.0003	17	15/2	0	0.75
16S (347/405 bp)	**Arc**	21	2	0.095 ± 0.084	0.00178 ± 0.00027	1	—	0	0.03	**Arc**	21	1	0	0	0	—	0	0
	Bar**	35	4	0.518 ± 0.077	0.00165 ± 0.00030	2	—	1	0.17	**GoM**	15	7	0.848 ± 0.065	0.00301 ± 0.00046	6	—	0	0.30
	**Ant**	30	2	0.434 ± 0.070	0.00751 ± 0.00121	6	—	0	0.77	**Ant**	22	1	0	0	0	—	0	0
	Tot**	86	7	0.758 ± 0.00036	0.00663 ± 0.00053	8	—	1	0.67	**Tot**	58	9	0.724 ± 0.038	0.00318 ± 0.00018	8	—	0	0.32
CytB (304/217 bp)	**Arc**	16	3	0.642 ± 0.067	0.00249 ± 0.00042	2	1/1	0	0.15	**Arc**	20	2	0.521 ± 0.042	0.0024 ± 0.00019	1	1/0	0	0.24
	**Bar**	25	5	0.690 ± 0.080	0.00866 ± 0.00137	8	5/2*	0	0.54	**GoM**	10	3	0.511 ± 0.164	0.00256 ± 0.00094	2	1/1	0	0.26
	**Ant**	14	3	0.582 ± 0.092	0.01898 ± 0.00232	11	10/1	0	1.23	**Ant**	20	1	0	0	0	—	0	0
	**Tot**	55	10	0.869 ± 0.019	0.01633 ± 0.00173	18	14/4*	0	1.04	**Tot**	50	6	0.753 ± 0.036	0.01509 ± 0.00052	8	7/1	0	1.55
28S (560/589 bp)	**Arc**	26	5	0.751 ± 0.051	0.00198 ± 0.00025	2	—	0	0.20	**Arc**	24	3	0.424 ± 0.112	0.00078 ± 0.00023	2	—	0	0.08
	**Bar**	46	5	0.670 ± 0.040	0.00150 ± 0.00015	3	—	0	0.15	**GoM**	28	2	0271 ± 0.099	0.00046 ± 0.00017	1	—	0	0.04
	**Ant**	44	2	0.169 ± 0.071	0.00121 ± 0.00051	4	—	0	0.12	**Ant**	28	1	0	0	0	—	0	0
	**Tot**	116	9	0.604 ± 0.047	0.0018 ± 0.00025	9	—	0	0.18	**Tot**	80	4	0.675 ± 0.025	0.00151 ± 0.00012	3	—	0	0.15

Arc = Arctic, Bar = Barbados Trench, Ant = Antarctic, GoM = Gulf of Mexico, n = sample size (for 28S this is given as total number of sequences; two per specimen), k = number of different haplotypes, H = haplotype diversity, π = nucleotide diversity, SD = standard deviation, P = polymorphic sites, S/N = synonymous/non-synonymous substitutions, In-Dels = insertions/deletions, M = mean p-distance. *One substitution was outside the reading frame, and it could thus not be determined if it was synonymous or not. **The partition contains an in-del, which was excluded by DnaSP. This position is therefore not included in the calculations of haplotype diversity and nucleotide diversity, but all other statistics include the gap as a 5th state, and the sequences including a gap as a distinct haplotype.

Haplotype networks were generated in TCS v1.21^38^ with gaps as a fifth state, drawn in PopART (http://popart.otago.ac.nz), and final graphical adjustments were made in Adobe Illustrator CS6 (Adobe Systems, San Jose, CA, USA). Genetic diversity within each population (Arctic, Gulf of Mexico/Barbados and Antarctic) was calculated in DnaSP^39^. Pairwise p-distance (uncorrected p) matrixes for COI haplotypes and within group mean p-distances were calculated in MEGA7^40^. GenAlEx was used to perform AMOVA (analysis of molecular variance) and to calculate pairwise PhiPT values^41^. A permutation test with 999 permutations was performed for both the AMOVA and pairwise PhiPT values to test if the observed values were significantly different from the null distribution.

### Phylogenetic analyses and species delimitation

Species delimitation for *S. contortum* and *N. lokii* was performed under the multi-species coalescent model (MSC) using STACEY^42^, a package for BEAST2^43^. Firstly, species delimitation for both species were performed without any outgroups, using the same four markers as in the population genetic analyses. Secondly, a species tree was reconstructed for *Nicomache*, also using STACEY, based on COI, 16S, 28S and 18S. This analysis included three additional species of *Nicomache* (*N. lumbricalis*, *N. minor* and *N. quadrispinata*), and also one species of the closely related genus *Petaloproctus* (*Petaloproctus tenuis*), which was used to root the tree. Species tree reconstruction under the multispecies coalescent model requires sequences from multiple specimens per species, and this was not available for other species of *Sclerolinum* apart from *S. contortum*, so therefore no phylogeny was reconstructed for this genus.

Datasets for species delimitation and phylogenetic reconstruction were assembled so that all haplotypes and combinations of haplotypes present in each population were represented (see Supplementary Tables 2–4. For example, if two individuals had the same 16S haplotype, but different COI haplotypes, both combinations were included. For 28S each haplotype of the diploid genotype was considered independently, so for heterozygote individuals a “mock individual” was generated for each haplotype of 28S (named with the specimen ID followed by A or B). The only haplotype not included in the species delimitation/phylogenetic datasets was the COI haplotype E for *S. contortum*, because this was the only gene sequenced for that specimen (see Supplementary material, Table S4). A concatenated matrix of all genes was generated using Sequence Matrix^44^, which automatically generates empty sequences of question marks for individuals with missing data for one or more genes. Substitution saturation was tested for the third position of COI and CytB using the Xia method implemented in DAMBE6^45^, but no saturation was detected. The best partition scheme and the best fitting model of evolution for each partition was found using Partition Finder v2.1.1 with the greedy algorithm and PhyML^46,47^. The input file was partitioned with each gene and each codon position of COI and CytB as separate partitions. The best-fit partition schemes and site models for each dataset can be found in Supplementary Table S5. Due to statistical concerns regarding the co-estimation of the gamma and invariant-site parameters (discussed in the RAxML manual^48^) we chose to use only the gamma model for rate heterogeneity for the partitions where Partition Finder suggested to use both.

STACEY incorporates species delimitation and phylogenetic reconstruction in the same MCMC run, and therefore it does not require *a priori* species designations. All specimens were defined as different species in the initial settings, leaving delimitation to the analysis. For the phylogenetic analysis of *Nicomache* the tree was rooted by defining the ingroup (all specimens of *Nicomache* spp.) as monophyletic. For all analyses, the site models were linked and substitution models defined as suggested by Partition Finder, but the phylogenetic analysis of *Nicomache* would not reach convergence with this model. Therefore, the model for this analysis was simplified by setting all site models to HKY, but with site heterogeneity models as before. For all analyses, the clock models were unlinked, while the tree model was linked for all the mitochondrial partitions as these are inherited as a unit, and thus expected to share the same phylogenetic history. It can be argued that 28S and 18S are not completely independent either, as these genes are located close together in the nuclear genome, and therefore we ran two separate analyses for the phylogeny of *Nicomache*, with the tree models for these genes linked and unlinked. For comparison, species delimitation was also performed for the phylogenetic analyses of *Nicomache*. Priors and settings for the STACEY analyses can be found in Supplementary Methods.

### Data availability

The datasets analyzed during the current study are available in a Figshare repository: 10.6084/m9.figshare.5746755.

## Results

### Population genetic analyses

**Figure 2:**
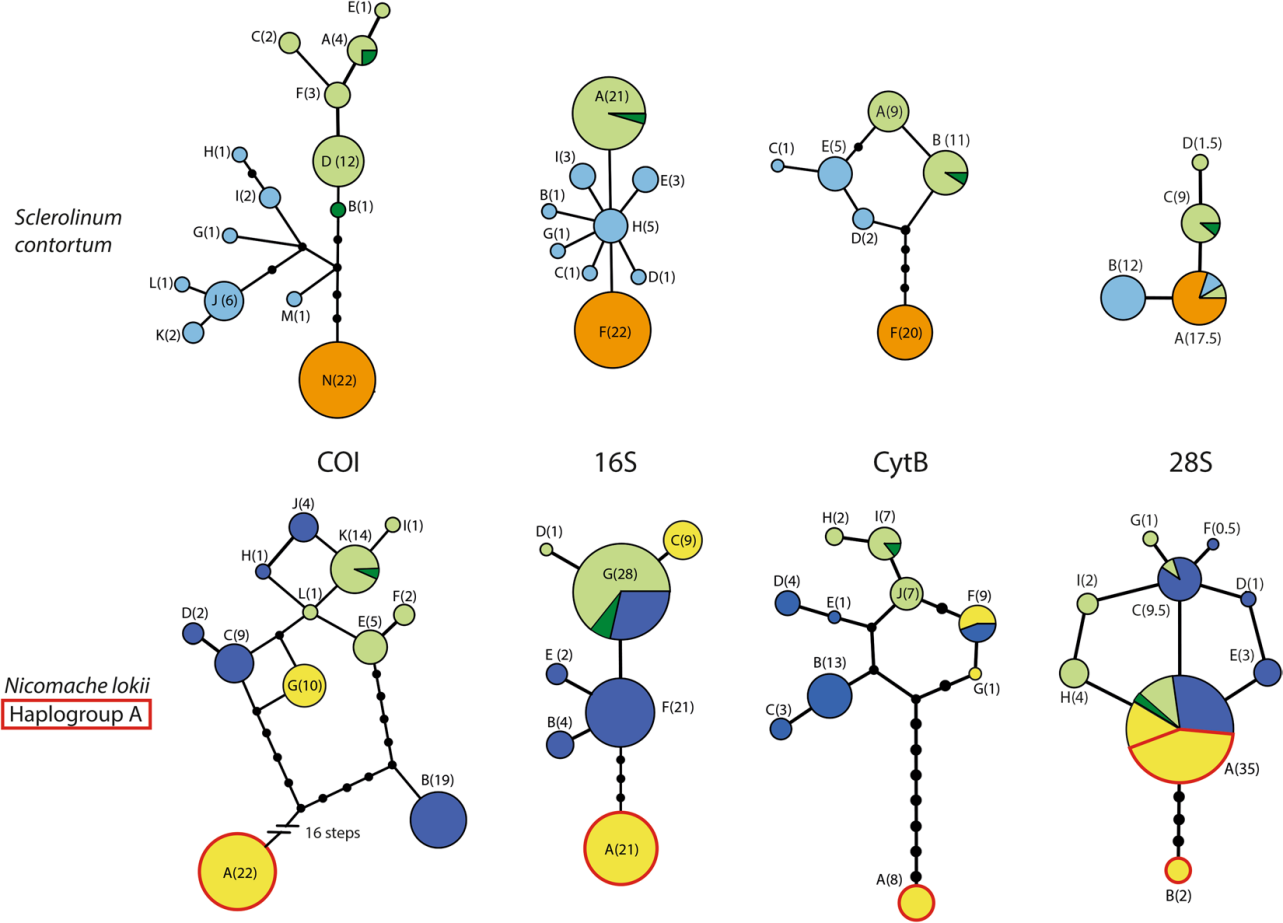
TCS haplotype networks of COI, 16S, CytB and 28S of *Sclerolinum contortum* and *Nicomache lokii* Each line represents one mutation, and black dots represents missing haplotypes. The size of the circles is proportional to the sample size of the haplotype, and haplotype designation and sample size (in brackets) is also indicated for each haplotype. For 28S two sequences were included per specimen, but the size of the circles is scaled to represent number of specimens, and the number in brackets is also scaled in the same way (number of sequences divided by two). Haplotypes are colored according to location, and corresponds to the colors in Fig. 1: Loki’s Castle – light green, HMMV – dark green, GoM – light blue, Barbados Trench – dark blue, Hook Ridge – orange, eastern Scotia Sea – yellow. For *Nicomache lokii*, haplogroup A is highlighted with a red outline.

Between 55–116 sequences per gene for *N. lokii* and 18–50 for *S. contortum* were included in the population genetic analyses (counting two sequences per individual for 28S, see Table 2). Alignment lengths varied between 217 bp (CytB for *S. contortum*) to 656 bp (COI for *Nicomache lokii*, see Table 2). COI was the most variable marker in both datasets, with the highest number of haplotypes and polymorphic sites (see Table 2 and Fig. 2). The highest pairwise p-distance for COI in *S. contortum* was 1.7%, between haplotype E from Loki’s Castle and haplotype H from GoM (see Supplementary Table S6). For *N. lokii* the highest pairwise p-distance was 4.1% between haplotype A in the Antarctic and haplotypes F and I from Loki’s Castle and haplotype J in the Barbados Trench (see Supplementary Table S7). Not considering haplotype A, the highest COI p-distance for *N. lokii* was 1.9% between haplotype B from the Barbados Trench and haplotypes I from Loki’s Castle and J from the Barbados Trench.

The network topology for 16S of *S. contortum* was very similar to that found previously by Georgieva *et al*.^25^ for COI, but with lower variation (see Fig. 2). For COI, CytB and 28S of *S. contortum*, the genetic diversity was very similar for the Arctic and the GoM populations, while the lowest diversity was found in the Antarctic population, which had only a single haplotype for all markers (see Table 2). For 16S the highest genetic diversity was found in the GoM population (Table 2). Both network topology and pairwise PhiPT values for COI and 16S showed a pattern of decreasing genetic similarity with increasing geographic distance, as both the Arctic and Antarctic populations were more genetically similar to the GoM population than to each other (Table 3). However, for COI and CytB pairwise PhiPTs were lower between the GoM and Arctic populations than between the GoM and Antarctic populations (Table 3). For 28S the variation in *S. contortum* was low in all populations with only 4 haplotypes in total, and one of these were shared between all populations (Fig. 2). Pairwise PhiPTs for 28S were similar for all populations (Table 3). Results from the AMOVA shows that most of the genetic variation in *S. contortum* was between populations for all markers (between 82–93%, see Table 4).

**Table 3:** Pairwise PhiPT values calculated in GenAlEx.

	*Nicomache lokii*			*Sclerolinum contortum*		
	Bar/Arc	Bar/Ant	Ant/Arc	GoM/Arc	GoM/Ant	Ant/Arc
COI	0,460***	0,560***	0,672***	0,720***	0,862***	0,909***
16S	0,562***	0,563***	0,610***	0,687***	0,680***	1,000***
CytB	0,497***	0,499***	0,602***	0,797***	0,969***	0,952***
28S	0,193**	0,249***	0,310***	0,843***	0,846***	0,850***

Significance of PhiPT values calculated with 999 permutations is shown. *P < 0.05, **P < 0.01, ***P < 0.001.

**Table 4:** Results of the AMOVA performed in GenAlEx.

*Nicomache lokii*		df	SS	MS	Est. Var.	%	PhiPT
COI	Among Pops	2	262,611	131,305	4,338	59%	
	Within Pops	87	263,834	3,033	3,033	41%	
	Total	89	526,444		7,371	100%	0,589***
16S	Among Pops	2	49,159	24,580	0,854	58%	
	Within Pops	83	50,352	0,607	0,607	42%	
	Total	85	99,512		1,460	100%	0,585***
CytB	Among Pops	2	59,249	29,624	1,592	53%	
	Within Pops	52	74,788	1,438	1,438	47%	
	Total	54	134,036		3,030	100%	0,525***
28S	Among Pops	2	21,141	10,571	0,483	25%	
	Within Pops	55	81,566	1,483	1,483	75%	
	Total	57	102,707		1,966	100%	0,246***
***Sclerolinum contortum***		**df**	**SS**	**MS**	**Est. Var**.	**% Var**.	**PhiPT**
COI	Among Pops	2	101,588	50,794	2,387	84%	
	Within Pops	62	27,304	0,440	0,440	16%	
	Total	64	128,892		2,828	100%	0,844***
16S	Among Pops	2	28,139	14,070	0,729	82%	
	Within Pops	55	8,533	0,155	0,155	18%	
	Total	57	36,672		0,884	100%	0,825***
CytB	Among Pops	2	72,750	36,375	2,264	93%	
	Within Pops	47	7,450	0,159	0,159	7%	
	Total	49	80,200		2,422	100%	0,935***
28S	Among Pops	2	52,993	26,496	1,965	84%	
	Within Pops	37	13,357	0,361	0,361	16%	
	Total	39	66,350		2,326	100%	0,845***

Significance of PhiPT values calculated with 999 permutations is shown. *P < 0.05, **P < 0.01, ***P < 0.001.

The patterns of genetic diversity for *N. lokii* were more complex. The number of haplotypes was similar between the Arctic and Barbados Trench populations (same number of haplotypes for COI and 28S, higher number in Barbados Trench for 16S and CytB), while the Antarctic population had fewer haplotypes for most of the markers (see Table 2). However, for the Barbados Trench and Antarctic populations, the haplotypes did not cluster together in the haplotype networks, and this was especially apparent in the mitochondrial markers (Fig. 2). The high level of divergence between mitochondrial haplotypes in the Barbados Trench and Antarctic populations was also reflected in the measures of nucleotide diversity (π) and mean p-distance within populations (Table 2). In the Antarctic population, there were only two haplotypes for COI, 16S and 28S, while for CytB there was also a third haplotype which was one basepair different from haplotype F (Fig. 2). For all the mitochondrial markers one of the Antarctic haplotypes was closely related to haplotypes from the Arctic or the Barbados Trench (one or two mutation steps different in the networks, see Fig. 2), while the other haplotype (haplotype A for COI, 16S and CytB) was very different. For COI haplotype A was 22 mutation steps away from the nearest haplotypes (either haplotype G from the Antarctic or haplotype B from the Barbados Trench), which corresponds to 3.5% genetic distance (see Supplementary material, Table S6 for pairwise p-distances between COI haplotypes). However, for 28S the Antarctic group of specimens with the most divergent mitochondrial haplotypes (henceforth referred to as haplogroup A) had two haplotypes, and one of these (28S haplotype A) is shared with the remaining Antarctic specimens, and the other populations (Fig. 2). The two haplotypes for 28S found in the Antarctic population were found both as homozygotes (14 individuals homozygotic for A and one individual homozygotic for B) and heterozygotes (two individuals). Haplogroup A co-occurs with the second Antarctic lineage of *N. lokii* at both E2 and E9, while only the second lineage was present at Kemp Caldera. However, only two individuals were sequenced from Kemp Caldera, so it cannot be excluded that lineage A would be found there with further sampling.

Pairwise PhiPT values for *N. lokii* were lower than those found for *S. contortum*, with values ranging between 0.460–0.672 for the mitochondrial markers, and 0.193–0.310 for 28S (Table 3). However, much like the pattern found in *S. contortum*, the highest PhiPT values were found between the Arctic and Antarctic populations for all markers, and for COI and 28S the lowest values were found between the Arctic and Barbados Trench populations. The high genetic variation within populations of *N. lokii* was reflected in the AMOVA results (Table 4). For the mitochondrial markers, between 41–47% of the variation was found within populations, while for 28S the number was even higher, with 75% of the variation found within populations. This was much higher than the values found in *S. contortum*, where within population variation accounted for between 7–18% of the variation (Table 4).

### Phylogenetic analyses and species delimitation

Based on the criteria outlined in Methods, 21 specimens were included in the species delimitation analysis of *S. contortum*, of which one specimen was a “mock specimen” (see Supplementary Table S4). 27 specimens were included in the species delimitation analysis of *N. lokii*, of which three were “mock specimens” (see Supplementary Table S2). The species delimitation analysis for *S. contortum* did not recover any well supported clustering schemes. The most common scheme in the posterior distribution of trees (four species, with one species each in the Arctic and Antarctic, and two species in the Gulf of Mexico) was only found in 3% of the trees. In all species delimitation analyses of *N. lokii*, the clustering scheme that received the highest support (49% in the analysis without outgroups, 71% in the analysis with outgroups and unlinked nuclear markers, and 68% in the analysis with outgroups and with linked nuclear markers), recovered *N. lokii* from the Arctic, the Barbados Trench and the Antarctic (including haplogroup A) as a single cluster. The second-best clustering scheme in all analyses (13% in the analysis without outgroups, 25% in the analysis with outgroups and unlinked nuclear markers, and 30% in the analysis with outgroups and with linked nuclear markers) recovered the same cluster as above, except haplogroup A, which was recovered as a separate cluster. All remaining clustering schemes were found in less than 2% of the trees.

**Figure 3:**
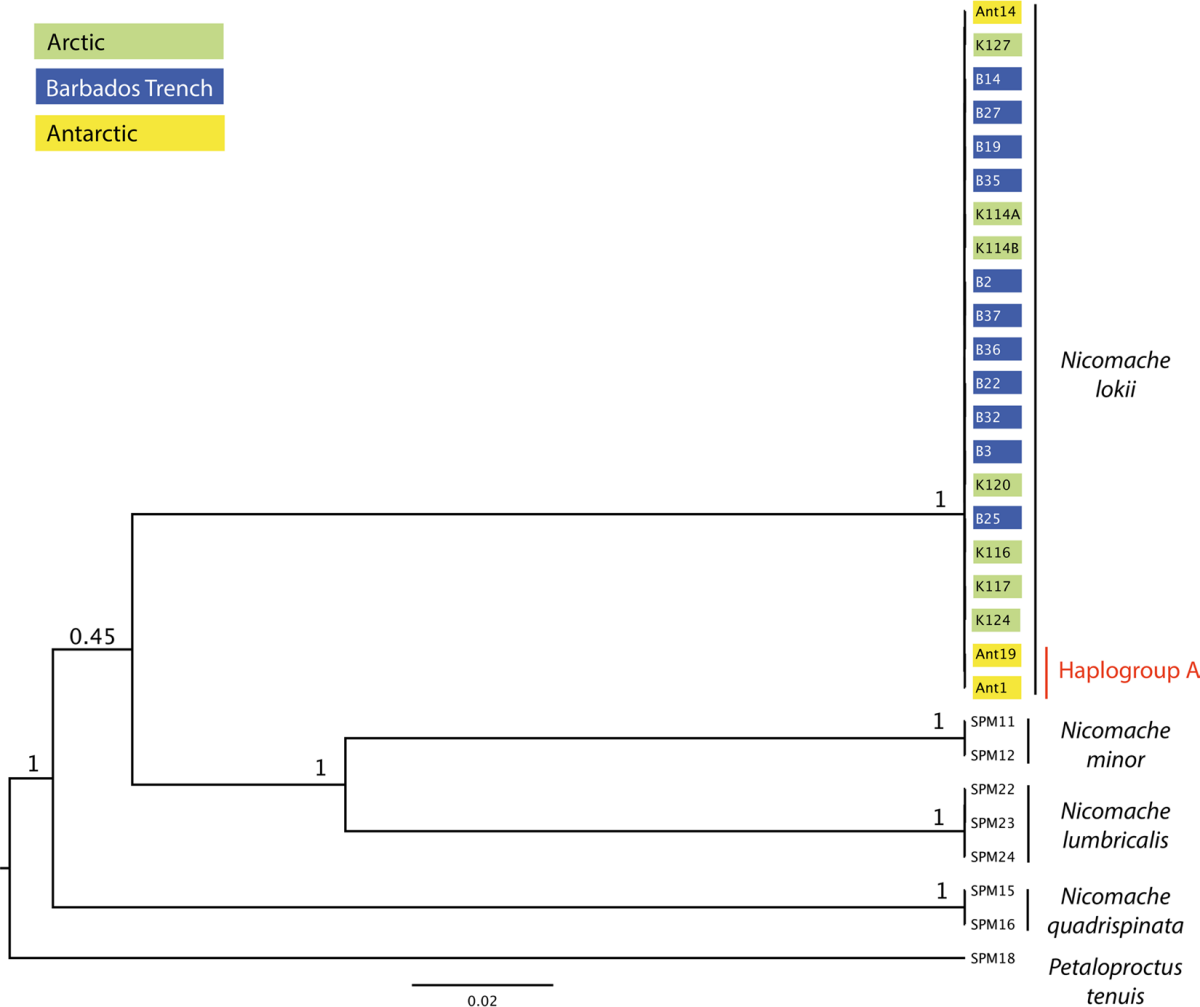
Species tree of *Nicomache* spp. with *Petaloproctus tenuis* as outgroup. Node values represent posterior probabilities and node heights are median heights. The phylogeny was inferred under the multispecies coalescent model in BEAST2 using the STACEY package for species delimitation, and with the tree models for 28S and 18S linked.

In total 102 sequences were included in the phylogenetic analyses of *Nicomache*, all newly produced for this study (see Supplementary Table S2). Eight specimens were included from the Arctic, eleven specimens from the Barbados Trench and three specimens from the Antarctic (of which two were from haplogroup A). 18S was sequenced for one specimen from the Arctic, four specimens from the Barbados Trench and three specimens from the Antarctic, and showed no variation between these populations. The results were mainly the same for the phylogenetic analyses with the tree models for the nuclear markers 18S and 28S linked and unlinked. The analysis with linked 18S and 28S tree models converged faster (1 × 10^9^ generations versus 3 × 10^9^ generations), and gave somewhat higher node support, so only the tree from this analysis is presented here (Fig. 3; see Supplementary material, Figure S1 for the tree from the analysis with unlinked nuclear markers). The phylogenetic reconstruction recovered *N. lokii* from the Arctic, Barbados Trench and the Antarctic as a monophyletic group with maximum support (Fig. 3). *N. minor* and *N. lumbricalis* are recovered together with high support, but the position of *N. quadrispinata* relative to the other *Nicomache* species is unresolved (Fig. 3).

## Discussion

The results presented here support that all the populations of *Nicomache lokii* are conspecific, extending the range of this species all the way from the Arctic to the Antarctic. Although species delimitation analyses did not provide any evidence in support of or against the hypothesis of conspecificity of the *Sclerolinum contortum* populations, the levels of genetic divergence between populations is low in all markers, both mitochondrial and nuclear, which corroborates the findings of Georgieva *et al*.^25^. We thereby conclude that the conspecificity of the populations of *S. contortum* is still supported. Although the geographic ranges of *N. lokii* and *S. contortum* are, to the best of our knowledge, the widest recorded of any species obligate to chemosynthesis-based ecosystems, wide ranges are not unusual among annelids from CBEs. A polynoid species from the Longqi vent field in the Indian Ocean is also found on the East Scotia Ridge (a range of ∼6000 km), and a phyllodocid species (*Hesiolyra* cf. *bergi*) is potentially shared between hydrothermal vents at Longqi, on the East Pacific Rise and the Mid Atlantic Ridge^49^. Two annelid species in the genus *Archinome* (Amphinomida) appear to be shared between CBEs in several ocean basins, but one of these (*Archinome tethyana*) has only been sequenced from the Mid-Atlantic Ridge and the other (*Archinome jasoni*) shows strong genetic structure between the West-Pacific and Indian Ocean sites^50^. Future efforts with genetic analyses of annelids from CBEs around the world is necessary to elucidate how common widespread distributions across several oceans are.

In the case of *N. lokii*, it seems likely that the presence of two sympatric but very distinct mitochondrial lineages of *N. lokii* in the Antarctic, one of which has very similar haplotypes to those found in the Arctic and Barbados Trench, is the result of two independent colonization events. The genetic divergence between them could be the result of Haplogroup A having evolved independently in the Antarctic for a period of time before the arrival of the second lineage, possibly due to infrequent long-distance dispersal events such as those inferred to occur in the West-Pacific^51^. An alternative explanation is that Haplogroup A dispersed to the Antarctic from an as of yet unknown population of *N. lokii* that is isolated from the Arctic and Barbados Trench populations. When two divergent lineages come into secondary contact, they will, depending on how far along they are in the speciation process, either remain distinct or merge into one interbreeding population^52^. The presence of a shared 28S haplotype between these two lineages could be the result of either incomplete lineage sorting, or recent geneflow. Species delimitation results support that Haplogroup A in the Antarctic belongs to *N. lokii*, although this lineage shows levels of COI divergence usually considered to be in the interspecific range^53^. Species delimitation under the multispecies coalescent model assumes that all genetree discordance is caused by incomplete lineage sorting, and the analysis is known to be sensitive to violations of the model assumptions such as population structure or geneflow between species^54,55^. Simulation studies indicate that STACEY is more sensitive to the latter scenario, and may lump species if there has been recent geneflow between two distinct lineages due to secondary contact^54^. Thus, it is possible that the species delimitation results could have been confounded if the two distinct Antarctic lineages are a result of secondary contact between two lineages with incomplete reproductive barriers. To test these hypotheses and resolve whether Haplogroup A may constitute a distinct species from *N. lokii* would require a higher sampling of more variable nuclear markers.

The Antarctic populations of both *N. lokii* and *Sclerolinum contortum* show very low genetic diversity compared to the central Atlantic and Arctic populations. There are several possible explanations for this low diversity, such as recent founder events, population bottlenecks or selective sweeps. It is curious, however, that the same low diversity is evident in both species, and in *N. lokii* it is evident in both lineages, and across several sampling localities in the eastern Scotia Sea. A population genetic analysis of three other species (a kiwaid squat lobster, a peltospirid gastropod and a leptodrilid limpet) from the eastern Scotia Sea hydrothermal vents did not show a similar depressed genetic diversity^56^, which excludes the possibility of a local event that affected all of the vent fauna. Both *S. contortum* and *N. lokii* are able to reproduce asexually by fragmentation and regrowth^28,57^, which may explain low levels of genetic diversity in one locality, as the sampled specimens may be clonal individuals. This is a plausible explanation for the low genetic diversity in *S. contortum*, as all of the sequenced individuals in the present study is from the same sampling locality^25^.

The pairwise PhiPT values (Table 3) for both *S. contortum* and *N. lokii* could indicate greater isolation of the Antarctic population compared to the Arctic and central populations (GoM and Barbados Trench). Apart from the geographic distance, further isolation of the Antarctic populations could be caused by the Antarctic Circumpolar Current, which presents a barrier at around 50°S. The benthic fauna of the Antarctic shows high degrees of endemism, but the isolation is less pronounced in the deep sea than at shelf depths^58^. For *N. lokii* the high PhiPTs could be an effect of the very divergent Haplogroup A, and the conspecificity of this lineage should be further assessed before making any conclusions based on statistics including this lineage. It is also possible that the low diversity in the Antarctic populations of both *N. lokii* and *S. contortum* are inflating the pairwise PhiPT values in comparisons including the Antarctic populations. PhiPT is calculated as the proportion of the variance among populations, relative to the total variance (within population variance + between population variance). Thus, lower within population variation will lead to higher PhiPT values.

Although our results support that both *S. contortum* and *N. lokii* have very wide distributions ranging from the Arctic to the Antarctic, the lack of shared haplotypes in COI and high PhiPTs between all populations indicates that present-day levels of geneflow are low. However, the existence of unsampled populations between the currently known populations is highly likely, and sampling gaps can give a false impression of isolation^59^. *Nicomache* sp. is known from the hydrothermal vents of the Mid-Cayman Spreading Center^60^, and comparison of a COI sequence from that locality (GenBank accession number KJ566962) with our dataset shows that it is 1.5% different from the most similar haplotype of *N. lokii* (haplotype B from the Barbados Trench). This is within the range of COI-variation considered to be intraspecific for *N. lokii* in this study. The Pacific species *Nicomache arwidsoni* has been recorded from the Logatchev vent field on the mid-Atlantic ridge^61^, but given the geographic distribution of *N. lokii* presented here it is possible that this population could also be *N. lokii*. In addition, there are both unsampled and probably also unrecorded cold seeps along the continental margins of the Atlantic which could provide suitable habitats for *N. lokii* and *S. contortum*.

Although genetic variation was consistently lower in *S. contortum* than *N. lokii*, the haplotype networks of *S. contortum* showed a much stronger geographic structure, which could be interpreted as a higher degree of isolation. Two possible scenarios can be put forward to explain the differences in the degree of geographic structure between the two species: it could either be a result of a difference in dispersal ability between *S. contortum* and *N. lokii*, or of the more isolated placement of the GoM seeps compared to the Barbados Trench. Unfortunately, there is very little known about the reproductive biology of either species. *Sclerolinum contortum* has oval-shaped eggs of up to 430 μm in length and 110 μm in diameter^27^, while the eggs of *N. lokii* are disc-shaped and up to 240 μm in diameter. This size range is usually associated with lecithotrophic larvae, and this is the prevalent larval mode among the vestimentiferans, the sister group of *Sclerolinum*^9^. To disentangle the effects of geographic barriers and putative differences in dispersal capacity on the genetic connectivity it would be best to have samples of both species from the same localities, however this was not available for the present study. Given the geographic distance between the populations and lack of shared haplotypes, the low degree of geographic structure in the haplotype networks of *N. lokii* is peculiar. In the COI network, several of the Barbados Trench haplotypes were more similar to haplotypes from the Arctic, than to other haplotypes in the Barbados Trench. To evaluate possible explanations for this pattern would require a better knowledge of potential intermediate populations between the Arctic and the Barbados Trench. Possible asymmetries in geneflow and time of isolation could be inferred using Isolation with Migration models, but these models assume that there has been no geneflow between unsampled populations and the sampled populations under analysis^62^, which cannot reasonably be assumed for the current dataset.

Both *N. lokii* and *S. contortum* show an ability to inhabit a wide range of chemosynthesis-based ecosystems, including various kinds of hydrothermal vents and also cold seeps. *Sclerolinum contortum* is in addition able to inhabit sunken wood, which could provide stepping stones for dispersal between vents and seeps^63^. *Nicomache lokii* is able to occupy a wide depth range as well, with the sites sampled in the present study ranging from 1262 m at HMMV to 4930 m at Atalante East in the Barbados Trench. The low habitat-specificity of these two species may be part of the explanation for their wide distributions, as this increases the number of available habitat-patches. Despite the lack of habitat selectivity observed in both *N. lokii* and *S. contortum*, there may be within-habitat conditions that favor the establishment of both species together, or of each species individually. At Loki’s Castle both species were found in the diffuse flow area of the vent field^30,64^, and *N. lokii* was also found on the walls of black-smoker chimneys, but there they were much smaller in size^28^. Studies of the habitat of *S. contortum* at the Hook Ridge vents in the Antarctic indicates it may be limited to low-temperature areas (∼20 °C) at which sulphide flux is also lower^31^, which is approximately the same temperature it occupies at Loki’s Castle^23^. *Sclerolinum contortum* was found to be absent in parts of Hook Ridge influenced by fluid temperatures of up to 49 °C at which siliceous crusts had precipitated^31^, and higher temperatures combined with greater sulphide availability may be a possible explanation as to the absence of this species at the E2 and E9 Antarctic vent sites. Hydrothermal venting at Hook Ridge also appears to be largely ephemeral in nature and characterized by diffuse flow at low temperatures, which has been proposed as a reason for the absence of typical vent fauna^65^. Taxa better adapted to make use of resources below the sediment surface such as *S. contortum* may be favored at these vents, whereas *N. lokii* may be excluded from Hook Ridge for reasons of insufficient sulphide availability (and therefore insufficient food) near the sediment surface, and an inability to directly utilise sulphide at greater sediment depths. The presence of *N. lokii* on the Mid Cayman Spreading Center and putatively on the Mid-Atlantic Ridge, fits well with the hypothesis that this species is adapted to higher temperatures than *S. contortum*, which is not known from either of these vent sites. These observations support the view that environmental factors varying across different CBEs, such as fluid flux, may play a central role in determining the faunal composition of these habitats^66^.

## Conclusions

The results presented here support that both *Nicomache lokii* and S*clerolinum contortum* are distributed all the way from the Arctic to the Antarctic, giving them the widest recorded geographic range of any species obligate to chemosynthesis-based ecosystems, which is also supported by genetic data. In the Antarctic, there are two divergent mitochondrial lineages of *N. lokii*, which may be the result of two independent colonization events. However, the conspecificity of the most divergent lineage (Haplogroup A) to *N. lokii* should be reassessed with a higher number of more variable nuclear markers. *S. contortum* shows a higher degree of geographic structure in the genetic diversity than *N. lokii*, but whether this is due to intrinsic factors such as dispersal capacity or a reflection of different degrees of isolation of the sampled localities cannot be resolved with the present data. There are probably numerous unsampled populations of *S. contortum* and *N. lokii*, and future studies with more extensive sampling throughout the range of these species are needed to understand the genetic connectivity from the Arctic to the Antarctic. Observations on the environmental conditions inhabited by the two species indicate that *N. lokii* may be limited by flow intensity while *S. contortum* is probably able to tolerate lower flow conditions, but limited by temperature.

## Electronic supplementary material

Supplementary material
