# Supplementary material for: Genetic connectivity from the Arctic to the Antarctic: Sclerolinum contortum and Nicomache lokii (Annelida) are both widespread in reducing environments
Source: Sci Rep. 2018 Mar 19;8:4810. doi: 10.1038/s41598-018-23076-0 (PMC5859262; doi:10.1038/s41598-018-23076-0)
Supplement: Supplementary file 1 — Supplementary material [file 41598_2018_23076_MOESM1_ESM.docx]

Supplementary material for “Genetic connectivity from the Arctic to the Antarctic: *Sclerolinum contortum* and *Nicomache lokii* (Annelida) are both widespread in reducing environments.” by Eilertsen et al.

# Supplementary methods

## PCR reactions and protocols

PCR reactions contained 2.5 μL CoralLoad buffer from QIAGEN, 1 μL MgCl (QIAGEN, 25 mM), 2 μL dNTP (TaKaRa, 2.5 mM of each dNTP), 1 μL of each of the primers (10 μM solution), 0.15 mL TaKaRa HS Taq, 1 or 2 μL DNA extraction and ddH2O to make the total reaction volume 25 μL. For 28S 1 μL of Bovine Serum Albumin was added to the PCR reactions to increase PCR yield. In cases where the first PCR was unsuccessful, the DNA extraction was diluted by 10x with water before adding 1 μL of the diluted extraction to the PCR reaction. PCR cycling profiles were as follows: COI – 5 min at 95 °C, 5 cycles with 45 s at 95 °C, 45 s at 45 °C, and 1 min at 72 °C, followed by 35 cycles of 45 s at 95 °C, 45 s at 51 °C, and 1 min at 72 °C, and finally 10 min at 72 °C. 16S – 5 min at 95 °C, 35 cycles with 30 s at 95 °C, 30 s at 50 °C, and 1.5 min at 72 °C, and finally 10 min at 72 °C. CytB – 5 min at 94 °C, 6 cycles with 45 s at 94 °C, 45 s at 47 °C, and 1 min at 72 °C, followed by 36 cycles of 45 s at 94 °C, 45 s at 50 °C, and 1 min at 72 °C, and finally 8 min at 72 °C. 18S – 3 min at 94 °C, 35 cycles with 1 min at 94 °C, 1.5 min at 42 °C, and 2 min at 72 °C, and finally 7 min at 72 °C. 28S – 5 min at 94 °C, 40 cycles with 30 sec at 94 °C, 30 sec at 59 °C and 2 min at 72 °C, and finally 10 min at 72 °C.

## Priors and settings for STACEY analysis

For all analyses, ploidy was set to 1 for the mitochondrial markers and 2 for the nuclear markers. A strict molecular clock model was selected, and the prior for clock rate was set as a lognormal distribution with M=0 and S=1. The relative death rate was fixed to 0.5, the prior for the species growth rate was given a lognormal distribution with a mean (M) of 4.6 and standard deviation (S) of 2, and popPriorScale was modeled with a lognormal distribution with M=-7 and S=2. The remaining priors were left at the default. Multiple independent runs were performed for all analyses. For the species delimitation of *Nicomache* and *Sclerolinum* two independent runs were performed with 1 x 10^7^ generations and sampling every 50 000 generations. For the phylogenetic analyses of *Nicomache* each run was 5 x 10^8^ generations with sampling every 50 000 generations, and two independent runs were performed for the model with 28S and 18S linked and six runs for the model with 28S and 18S unlinked. All analyses were run on the CIPRES Science Gateway^1^. The log files were examined in Tracer v1.5 to check for convergence (ESS > 200 for combined runs)^2^. Runs were combined and burn-in (10% for each run) was removed using LogCombiner v2.4.4, and maximum clade credibility trees were generated in TreeAnnotator v2.4.4 (both in the BEAST2 package). All trees were converted to graphics using FigTree v1.4.0^3^ and final adjustments were made in Adobe Illustrator v16.0.4 (Adobe Systems, San Jose, CA, USA). The species delimitation results were calculated from the posterior distribution of trees (excluding burnin) using SpeciesDelimitationAnalyser^4^.

**Table S1.** PCR and sequencing primers.

|  | **Primer** | **Sequence 5′-3′** | **Direction** | **Source** |
| --- | --- | --- | --- | --- |
| **COI** | LCO1490 | GGTCAACAAATCATAAAGATATTGG | Forward | Folmer et al.^5^ |
|  | HCO2198 | TAAACTTCAGGGTGACCAAAAATCA | Reverse | Folmer et al.^5^ |
| **16S** | 16Sar-L | CGCCTGTTTATCAAAAACAT | Forward | Palumbi et al.^6^ |
|  | 16Sbr-H | CCGGTCTGAACTCAGATCACGT | Reverse | Palumbi et al.^6^ |
| **28S** | 28F5 | CAAGTACCGTGAGGGAAAGTTG | Forward | Passamaneck et al.^7^ |
|  | Po28R4 | GTTCACCATCTTTGGGGTCCCAAC | Reverse | Struck et al.^8^ |
| **CytB** | F424 | GGWTAYGTWYTWCCWTGRGGWCARAT | Forward | Boore and Brown^9^ |
|  | R876 | GCRTAWGCRAAWARRAARTAYCAYT  CWGG | Reverse | Boore and Brown^9^ |
| **18S** | 18e | CTGGTTGATCCTGCCAGT | Forward | Hillis and Dixon^10^ |
|  | 18L | GAATTACCGCGGCTGCTGGCACC | Reverse | Halanych et al.^11^ |
| Halanych et al.^11^ | 18F509 | CCCCGTAATTGGAATGAGTACA | Forward | Struck et al.^12^ |
|  | 18R | GTCCCCTTCCGCAATTYCTTTAAG | Reverse | Passamaneck et al.^7^ |
|  | 18F997 | TTCGAAGACGATCAGATACCG | Forward | Struck et al.^12^ |
|  | 18R1843 | GATCCAAGCTTGATCCTTCTGCAGGTTC  ACCTAC | Reverse | Struck et al.^13^ |

**Table S2.** Sampling data and GenBank accession numbers for specimens of *Nicomache lokii* included in the species delimitation analysis. Sequences marked with a * have accession numbers on the line above.

| **Seq ID** | | **Species** | **Locality** | **Lat** | **Lon** | **Depth (m)** | **COI** | **16S** | **CytB** | **28S** |
| --- | --- | --- | --- | --- | --- | --- | --- | --- | --- | --- |
| Ant10 | *Nicomache lokii* | Kemp Caldera Clam Road JC42 | - 59.86833 | - 28.3725 ­ | 1400 | MG975480 (G) | MG975527 (C) | MG975504 (F) | MG975550 (A) |  |
| Ant14 | | *Nicomache lokii* | E2 Cindy's Castle | -56.08833 | -30.31866 | 2646 | MG975481 (G) | MG975528 (C) | MG975505 (G) | MG975551 (A) |
| Ant15 | | *Nicomache lokii* | E2 Cindy's Castle | -56.08833 | -30.31866 | 2646 | MG975482 (A) | MG975529 (A) | MG975506 (A) | MG975552 (A) |
| Ant19 | | *Nicomache lokii* | E2 Crab City | -56.08833 | -30.31800 | 2641 | MG975483 (A) | MG975530 (A) | MG975507 (A) | MG975553 (B) |
| B3 | | *Nicomache lokii* | Barbados Trench, Atalante East | 13.82822 | -57.64472 | 4930 | MG975490 (B) | MG975537 (F) | MG975514 (C) | MG975560 (C) |
| B8 | | *Nicomache lokii* | Barbados Trench, Manon | 13.77773 | -57.54255 | 4742 | MG975495 (B) | MG975541 (F) | MG975518 (B) | MG975566 (C) |
| B11 | | *Nicomache lokii* | Barbados Trench, Atalante East | 13.82822 | -57.64472 | 4930 | MG975484 (B) | MG975531 (F) | MG975508 (C) | MG975554 (A) |
| B14 | | *Nicomache lokii* | Barbados Trench, Atalante East | 13.82822 | -57.64472 | 4930 | MG975485 (J) | MG975532 (B) | MG975509 (D) | MG975555 (D) |
| B19 | | *Nicomache lokii* | Barbados Trench, Atalante East | 13.82822 | -57.64472 | 4930 | MG975486 (J) | MG975533 (B) | MG975510 (D) | MG975556 (A) |
| B22 | | *Nicomache lokii* | Barbados Trench, Atalante East | 13.82822 | -57.64472 | 4930 | MG975487 (B) | MG975534 (E) | MG975511 (B) | MG975557 (E) |
| B25 | | *Nicomache lokii* | Barbados Trench, Atalante East | 13.82822 | -57.64472 | 4930 | MG975488 (C) | MG975535 (G) | MG975512 (F) | MG975558 (A) |
| B27 | | *Nicomache lokii* | Barbados Trench, Atalante East | 13.82822 | -57.64472 | 4930 | MG975489 (J) | MG975536 (B) | MG975513 (D) | MG975559 (E) |
| B35 | | *Nicomache lokii* | Barbados Trench, Atalante East | 13.82822 | -57.64472 | 4930 | MG975491 (H) | - | MG975515 (E) | MG975561 (C) |
| B36A | | *Nicomache lokii* | Barbados Trench, Atalante East | 13.82822 | -57.64472 | 4930 | MG975492 (C) | MG975538 (G) | MG975516 (F) | MG975562 (F) |
| B36B | | *Nicomache lokii* | Barbados Trench, Atalante East | 13.82822 | -57.64472 | 4930 | * | * | * | MG975563 (C) |
| B37 | | *Nicomache lokii* | Barbados Trench, Atalante East | 13.82822 | -57.64472 | 4930 | MG975493 (D) | MG975539 (G) | - | MG975564 (C) |
| B42 | | *Nicomache lokii* | Barbados Trench, Manon | 13.77773 | -57.54255 | 4742 | MG975494 (B) | MG975540 (F) | MG975517 (B) | MG975565 (A) |
| K114A | | *Nicomache lokii* | Lokis Castle | 73.5662 | 8.1585 | 2357 | MG975496 (L) | MG975542 (G) | MG975519 (J) | MG975567 (C) |
| K114B | | *Nicomache lokii* | Lokis Castle | 73.5662 | 8.1585 | 2357 | * | * | * | MG975568 (G) |
| K117 | | *Nicomache lokii* | Lokis Castle | 73.5662 | 8.1585 | 2357 | MG975497 (K) | MG975543 (G) | MG975520 (J) | MG975569 (H) |
| K120 | | *Nicomache lokii* | Lokis Castle | 73.5662 | 8.1585 | 2357 | MG975498 (K) | MG975544 (G) | MG975521 (I) | MG975570 (I) |
| K121 | | *Nicomache lokii* | Lokis Castle | 73.5662 | 8.1585 | 2357 | MG975499 (K) | MG975545 (G) | MG975522 (J) | MG975571 (I) |
| K123A | | *Nicomache lokii* | Lokis Castle | 73.5662 | 8.1585 | 2357 | MG975500 (K) | MG975546 (G) | MG975523 (I) | MG975572 (A) |
| K123B | | *Nicomache lokii* | Lokis Castle | 73.5662 | 8.1585 | 2357 | * | * | * | MG975573 (H) |
| K124 | | *Nicomache lokii* | Lokis Castle | 73.5662 | 8.1585 | 2357 | MG975501 (I) | MG975547 (D) | MG975524 (J) | MG975574 (A) |
| K127 | | *Nicomache lokii* | Lokis Castle | 73.5662 | 8.1585 | 2357 | MG975502 (E) | MG975548 (G) | MG975525 (I) | MG975575 (A) |
| SPM7 | | *Nicomache lokii* | Lokis Castle | 73.5662 | 8.1585 | 2357 | MG975503 (F) | MG975549 (G) | MG975526 (H) | - |

**­**

**Table S3.** Sampling data and GenBank accession numbers for specimens included in the phylogenetic analyses of *Nicomache* spp. For COI, 16S, and 28S haplotype designation of the sequence is indicated in brackets. Sequenced marked with a * have accession numbers in Table S3.

| **Seq ID** | **Species** | **Locality** | **Lat** | **Lon** | **Depth (m)** | **COI** | **16S** | **28S** | **18S** |
| --- | --- | --- | --- | --- | --- | --- | --- | --- | --- |
| SPM18 | *Petaloproctus tenuis* | Jan Mayen | 71,2997 | -5,7821 | 616 | MG975592 | - | MG975582 | MG975477 |
| SPM11 | *Nicomache minor* | Hvite Sea | 66,54331 | 33,18589 | - | MG975588 | MG975458 | MG975580 | MG975473 |
| SPM12 | *Nicomache minor* | Hvite Sea | 66,54331 | 33,18589 | - | MG975589 | MG975459 | - | MG975474 |
| SPM15 | *Nicomache quadrispinata* | Jan Mayen | 71,2998 | -5,7801 | 616 | MG975590 | MG975460 | MG975581 | MG975475 |
| SPM16 | *Nicomache quadrispinata* | Jan Mayen | 71,2998 | -5,7801 | 616 | MG975591 | MG975461 | - | MG975476 |
| SPM22 | *Nicomache lumbricalis* | Svalbard | 79,8233 | 12,0497 | 97 | MG975593 | MG975462 | - | MG975478 |
| SPM23 | *Nicomache lumbricalis* | Valsfjorden | 63,81325 | 9,64947 | 66 | MG975594 | - | MG975583 | - |
| SPM24 | *Nicomache lumbricalis* | Svalbard | 79,0246 | 11,6687 | 235 | MG975595 | MG975463 | - | MG975479 |
| Ant1 | *Nicomache lokii* | E2 JC42 | -56,08 | -30,31 | 2608 | MG975584 (A) | MG975455 (A) | MG975576 (A) | MG975465 |
| Ant14 | *Nicomache lokii* | E2 Cindy's Castle | -56,08833 | -30,31866 | 2646 | * | * | * | MG975466 |
| Ant19 | *Nicomache lokii* | E2 Crab City | -56,08833 | -30,318 | 2641 | * | * | * | MG975467 |
| B2 | *Nicomache lokii* | Barbados Trench, Atalante East | 13,82822 | -57,64472 | 4930 | MG975585 (C) | MG975456 (G) | MG975577 (C) | MG975468 |
| B3 | *Nicomache lokii* | Barbados Trench, Atalante East | 13,82822 | -57,64472 | 4930 | * | * | * | MG975469 |
| B14 | *Nicomache lokii* | Barbados Trench, Atalante East | 13,82822 | -57,64472 | 4930 | * | * | * | - |
| B19 | *Nicomache lokii* | Barbados Trench, Atalante East | 13,82822 | -57,64472 | 4930 | * | * | * | - |
| B22 | *Nicomache lokii* | Barbados Trench, Atalante East | 13,82822 | -57,64472 | 4930 | * | * | * | - |
| B25 | *Nicomache lokii* | Barbados Trench, Atalante East | 13,82822 | -57,64472 | 4930 | * | * | * | - |
| B27 | *Nicomache lokii* | Barbados Trench, Atalante East | 13,82822 | -57,64472 | 4930 | * | * | * | - |
| B32 | *Nicomache lokii* | Barbados Trench, Atalante East | 13,82822 | -57,64472 | 4930 | MG975586 (B) | MG975457 (F) | MG975578 (A) | - |
| B35 | *Nicomache lokii* | Barbados Trench, Atalante East | 13,82822 | -57,64472 | 4930 | * | - | * | MG975470 |
| B36 | *Nicomache lokii* | Barbados Trench, Atalante East | 13,82822 | -57,64472 | 4930 | * | * | * | MG975471 |
| B37 | *Nicomache lokii* | Barbados Trench, Atalante East | 13,82822 | -57,64472 | 4930 | * | * | * | - |
| K114A | *Nicomache lokii* | Lokis Castle | 73,5662 | 8,1585 | 2357 | * | * | * | MG975472 |
| K114B | *Nicomache lokii* | Lokis Castle | 73,5662 | 8,1585 | 2357 | * | * | * | - |
| K116 | *Nicomache lokii* | Lokis Castle | 73,5662 | 8,1585 | 2357 | MG975587 (K) | MG975464 (G) | MG975579 (A) | - |
| K117 | *Nicomache lokii* | Lokis Castle | 73,5662 | 8,1585 | 2357 | * | * | * | - |
| K120 | *Nicomache lokii* | Lokis Castle | 73,5662 | 8,1585 | 2357 | * | * | * | - |
| K124 | *Nicomache lokii* | Lokis Castle | 73,5662 | 8,1585 | 2357 | * | * | * | - |
| K127 | *Nicomache lokii* | Lokis Castle | 73,5662 | 8,1585 | 2357 | * | * | * | - |

**Table S4.** Sampling data and GenBank accession numbers for specimens included in the species delimitation analysis of *Sclerolinum contortum*. For COI, 16S, CytB and 28S haplotype designation of the sequence is indicated in brackets. Abbreviations: GoM - Gulf of Mexico, HMMV – Håkon Mosby Mud Volcano. Sequences marked with a * have accession numbers on the line above.

| **Seq ID** | **Species** | **Locality** | **Lat.** | **Lon.** | **Depth (m)** | **COI** | **16S** | **CytB** | **28S** |
| --- | --- | --- | --- | --- | --- | --- | --- | --- | --- |
| MG01 | *Sclerolinum contortum* | Hook Ridge, Antarctic | -62,1969 | -57,2975 | 1174 | KU214832 (N) | MG975436 (F) | MG975402(F) | MG975418 (A) |
| MG36/GoM1 | *Sclerolinum contortum* | Walker Ridge, GoM | 26.6833 | -91.65 | 1954 | KU214839 (K) | MG975437 (E) | - | MG975419 (B) |
| MG38/GoM3 | *Sclerolinum contortum* | Walker Ridge, GoM | 26.6833 | -91.65 | 1954 | KU214843 (L) | MG975438 (B) | MG975403 (E) | MG975420 (B) |
| MG40/GoM5 | *Sclerolinum contortum* | Walker Ridge, GoM | 26.6833 | -91.65 | 1954 | KU214841 (I) | MG975439 (I) | MG975404 (E) | MG975421 (B) |
| MG41/GoM6 | *Sclerolinum contortum* | Walker Ridge, GoM | 26.6833 | -91.65 | 1954 | KU214842 (H) | MG975440 (I) | MG975405 (D) | MG975422 (B) |
| MG42/GoM7 | *Sclerolinum contortum* | Walker Ridge, GoM | 26.6833 | -91.65 | 1954 | KU214842 (G) | MG975441 (C) | - | MG975423 (B) |
| MG44/GoM9 | *Sclerolinum contortum* | Walker Ridge, GoM | 26.6833 | -91.65 | 1954 | KU214845 (M) | MG975442 (H) | MG975406 (E) | MG975424 (A) |
| MG45/GoM10 | *Sclerolinum contortum* | Walker Ridge, GoM | 26.6833 | -91.65 | 1954 | KU214840 (J) | MG975443 (H) | MG975407 (C) | - |
| MG46/GoM11 | *Sclerolinum contortum* | Walker Ridge, GoM | 26.6833 | -91.65 | 1954 | KU214840 (J) | MG975444 (D) | MG975408 (E) | MG975425 (B) |
| MG48/GoM13 | *Sclerolinum contortum* | Walker Ridge, GoM | 26.6833 | -91.65 | 1954 | KU214840 (J) | MG975445 (H) | MG975409 (E) | MG975426 (B) |
| MG49/GoM14 | *Sclerolinum contortum* | Walker Ridge, GoM | 26.6833 | -91.65 | 1954 | KU214840 (J) | MG975446 (G) | - | MG975427 (A) |
| MG52/GoM17 | *Sclerolinum contortum* | Walker Ridge, GoM | 26.6833 | -91.65 | 1954 | KU214841 (I) | MG975447 (I) | MG975410 (D) | MG975428 (B) |
| S10 | *Sclerolinum contortum* | Lokis Castle | 73.5662 | 8.1585 | 2357 | KU214834 (D) | MG975448 (A) | MG975411 (B) | MG975429 (C) |
| S2 | *Sclerolinum contortum* | Lokis Castle | 73.5662 | 8.1585 | 2357 | KU214834 (D) | MG975449 (A) | MG975412 (B) | MG975430 (A) |
| S23 | *Sclerolinum contortum* | Lokis Castle | 73.5662 | 8.1585 | 2357 | KU214836 (C) | MG975450 (A) | MG975413 (A) | - |
| S28 | *Sclerolinum contortum* | HMMV | 71.9975– 71.9999 | 14.7329– 14.7316 | 1262 | KU214838 (B) | MG975451 (A) | MG975414 (B) | MG975431 (C) |
| S31 | *Sclerolinum contortum* | Lokis Castle | 73.5662 | 8.1585 | 2357 | KU214837 (A) | MG975452 (A) | MG975415 (A) | MG975432 (C) |
| S4 | *Sclerolinum contortum* | Lokis Castle | 73.5662 | 8.1585 | 2357 | KU214834 (D) | MG975453 (A) | MG975416 (B) | MG975433 (D) |
| S9A | *Sclerolinum contortum* | Lokis Castle | 73.5662 | 8.1585 | 2357 | KU214833 (F) | MG975454 (A) | MG975417 (A) | MG975434 (C) |
| S9B | *Sclerolinum contortum* | Lokis Castle | 73.5662 | 8.1585 | 2357 | KU214833 (F) | * | * | MG975435 (A) |
| S20* | *Sclerolinum contortum* | Lokis Castle | 73.5662 | 8.1585 | 2357 | KU214835 (E) | - | - | - |

**Table S5**. Best fit partitions and site models as calculated by Partition Finder for the species delimitation and phylogenetic analyses.

|  | **Partition** | **Model** |
| --- | --- | --- |
| *Nicomache* species delimitation | 16S, COI_1stpos, COI_2ndpos, CytB_1stpos, CytB_2ndpos | HKY + I |
|  | 28S | HKY + I + G |
|  | COI_3rdpos, CytB_3rdpos | TRN + I |
| *Nicomache* phylogeny | 16S, COI_1stpos | GTR + I |
|  | 28S, 18S, CytB_2ndpos | TRN + I + G |
|  | COI_3rdpos | HKY + G |
| *Sclerolinum* species delimitiation | 16S, COI_2ndpos, COI_3rdpos, CytB_2ndpos, CytB_3rdpos | TRN + I |
|  | 28S, COI_1stpos, CytB_1stpos | HKY + I |
|  |  |  |

**Table S6.** Pairwise K2P distances between COI haplotypes of *Sclerolinum contortum*.

|  | **A** | **B** | **C** | **E** | **F** | **D** | **G** | **H** | **I** | **J** | **K** | **L** | **M** |
| --- | --- | --- | --- | --- | --- | --- | --- | --- | --- | --- | --- | --- | --- |
| **B** | 0.006 |  |  |  |  |  |  |  |  |  |  |  |  |
| **C** | 0.004 | 0.006 |  |  |  |  |  |  |  |  |  |  |  |
| **E** | 0.002 | 0.007 | 0.006 |  |  |  |  |  |  |  |  |  |  |
| **F** | 0.002 | 0.004 | 0.002 | 0.004 |  |  |  |  |  |  |  |  |  |
| **D** | 0.004 | 0.002 | 0.004 | 0.006 | 0.002 |  |  |  |  |  |  |  |  |
| **G** | 0.013 | 0.009 | 0.009 | 0.015 | 0.011 | 0.011 |  |  |  |  |  |  |  |
| **H** | 0.015 | 0.011 | 0.011 | 0.017 | 0.013 | 0.013 | 0.009 |  |  |  |  |  |  |
| **I** | 0.011 | 0.007 | 0.007 | 0.013 | 0.009 | 0.009 | 0.006 | 0.004 |  |  |  |  |  |
| **J** | 0.011 | 0.007 | 0.007 | 0.013 | 0.009 | 0.009 | 0.006 | 0.007 | 0.004 |  |  |  |  |
| **K** | 0.013 | 0.009 | 0.009 | 0.015 | 0.011 | 0.011 | 0.007 | 0.009 | 0.006 | 0.002 |  |  |  |
| **L** | 0.013 | 0.009 | 0.009 | 0.015 | 0.011 | 0.011 | 0.007 | 0.009 | 0.006 | 0.002 | 0.004 |  |  |
| **M** | 0.011 | 0.006 | 0.007 | 0.013 | 0.009 | 0.007 | 0.007 | 0.009 | 0.006 | 0.006 | 0.007 | 0.007 |  |
| **N** | 0.011 | 0.009 | 0.011 | 0.013 | 0.013 | 0.011 | 0.011 | 0.013 | 0.009 | 0.009 | 0.011 | 0.011 | 0.007 |

**Table S7.** Pairwise K2P distances between COI haplotypes of *Nicomache lokii*

|  | **A** | **B** | **C** | **D** | **E** | **F** | **G** | **H** | **I** | **J** | **K** |
| --- | --- | --- | --- | --- | --- | --- | --- | --- | --- | --- | --- |
| **B** | 0.035 |  |  |  |  |  |  |  |  |  |  |
| **C** | 0.035 | 0.016 |  |  |  |  |  |  |  |  |  |
| **D** | 0.036 | 0.017 | 0.002 |  |  |  |  |  |  |  |  |
| **E** | 0.040 | 0.014 | 0.005 | 0.006 |  |  |  |  |  |  |  |
| **F** | 0.041 | 0.016 | 0.006 | 0.008 | 0.002 |  |  |  |  |  |  |
| **G** | 0.035 | 0.016 | 0.003 | 0.005 | 0.005 | 0.006 |  |  |  |  |  |
| **H** | 0.040 | 0.017 | 0.005 | 0.006 | 0.003 | 0.005 | 0.005 |  |  |  |  |
| **I** | 0.041 | 0.019 | 0.006 | 0.008 | 0.005 | 0.006 | 0.006 | 0.005 |  |  |  |
| **J** | 0.041 | 0.019 | 0.006 | 0.008 | 0.005 | 0.006 | 0.006 | 0.002 | 0.003 |  |  |
| **K** | 0.040 | 0.017 | 0.005 | 0.006 | 0.003 | 0.005 | 0.005 | 0.003 | 0.002 | 0.002 |  |
| **L** | 0.038 | 0.016 | 0.003 | 0.005 | 0.002 | 0.003 | 0.003 | 0.002 | 0.003 | 0.003 | 0.002 |

**Figure S1.** Species tree of *Nicomache* spp. with *Petaloproctus tenuis* as outgroup. Node values represent posterior probabilities and node heights are median heights. The phylogeny was inferred under the multispecies coalescent model in BEAST2 using the STACEY package for species delimitation, and with the tree models for 28S and 18S unlinked.


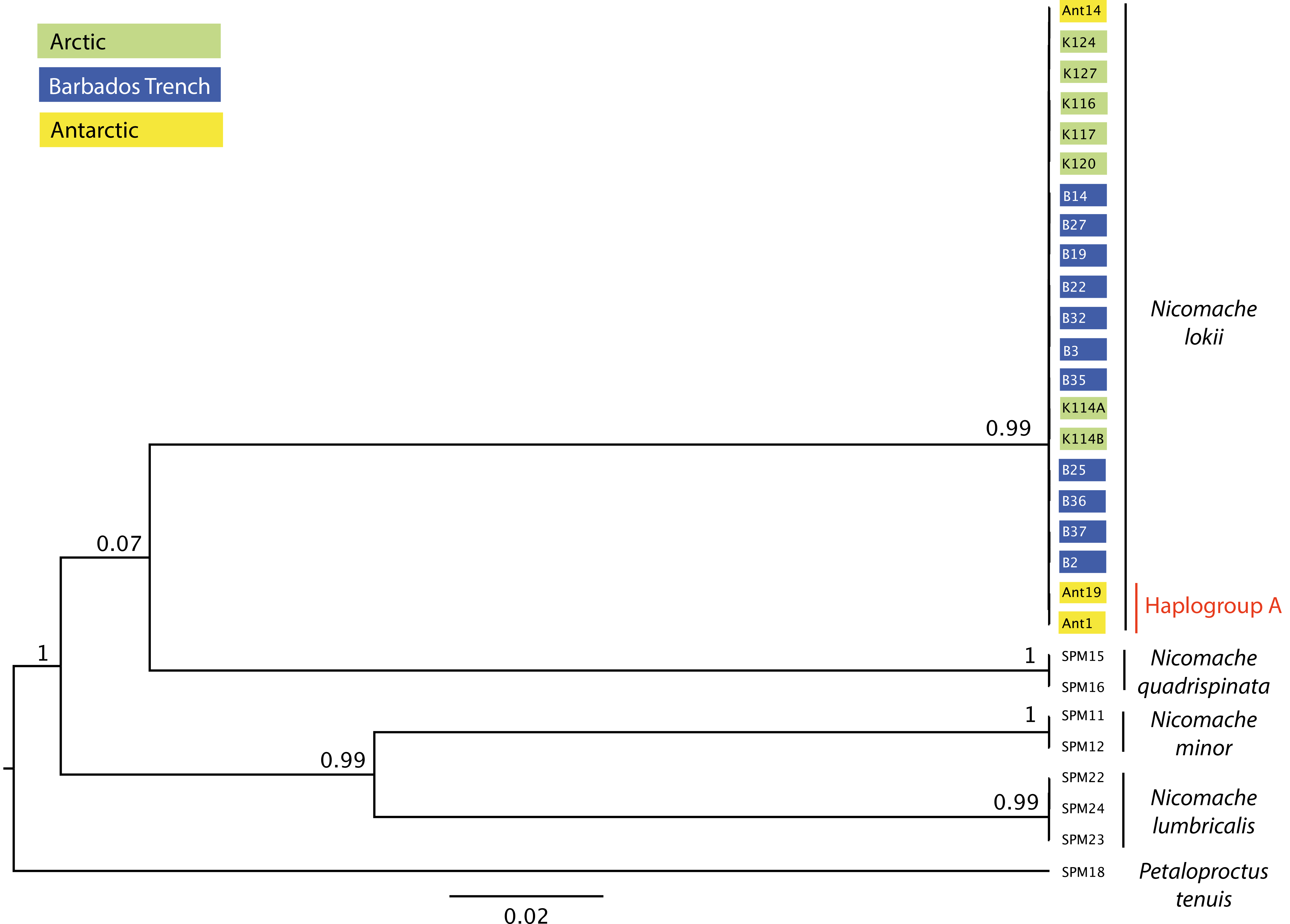


**References**

1 Miller, M. A., Pfeiffer, W. & Schwartz, T. Creating the CIPRES Science Gateway for inference of large phylogenetic trees. *Proceedings of the Gateway Computing Environments Workshop (GCE)*, 1-8 (2010).

2 Rambaut, A. & Drummond, A. J. *Tracer v1.5*, <<http://beast.bio.ed.ac.uk/Tracer>> (2009).

3 Rambaut, A. *FigTree. Version 1.4.0.*, <<http://tree.bio.ed.ac.uk/software/figtree>> (2012).

4 Jones, G. *Software page*, <<http://www.indriid.com/software.html>> (2017).

5 Folmer, O., Black, M., Hoeh, W., Lutz, R. & Vrijenhoek, R. DNA primers for amplification of mitochondrial cytochrome c oxidase subunit I from diverse metazoan invertebrates. *Mol. Mar. Biol. Biotechnol.* **3**, 294-299 (1994).

6 Palumbi, S. *et al.* *The Simple Fool's Guide to PCR*. (Special Publication, Department of Zoology, University of Hawaii, 1991).

7 Passamaneck, Y. J., Schander, C. & Halanych, K. M. Investigation of molluscan phylogeny using large-subunit and small-subunit nuclear rRNA sequences. *Mol. Phylogen. Evol.* **32**, 25-38, <http://dx.doi.org/10.1016/j.ympev.2003.12.016> (2004).

8 Struck, T. H., Pursche, G. & Halanych, K. M. Phylogeny of Eunicida (Annelida) and Exploring Data Congruence Using a Partition Addition Bootstrap Alteration (PABA) Approach. *Syst. Biol.* **55**, 1-20, <http://doi.org/10.1080/10635150500354910> (2006).

9 Boore, J. L. & Brown, W. M. Mitochondrial genomes of *Galathealinum*, *Helobdella*, and *Platynereis*: sequence and gene arrangement comparisons indicate that Pogonophora is not a phylum and Annelida and Arthropoda are not sister taxa. *Mol. Biol. Evol.* **17**, 87-106, <http://doi.org/10.1093/oxfordjournals.molbev.a026241> (2000).

10 Hillis, D. M. & Dixon, M. T. Ribosomal DNA: Molecular Evolution and Phylogenetic Inference. *The Quarterly Review of Biology* **66**, 411-453, <http://doi.org/10.2307/2831326> (1991).

11 Halanych, K. M. *et al.* Evidence from 18S ribosomal DNA that the lophophorates are protostome animals. *Science* **267**, 1641-1643 (1995).

12 Struck, T., Hessling, R. & Purschke, G. The phylogenetic position of the Aeolosomatidae and Parergodrilidae, two enigmatic oligochaete-like taxa of the “Polychaeta”, based on molecular data from 18SrDNA sequences. *Journal of Zoological Systematic and Evolutionary Research* **40**, 155–163, <http://doi.org/10.1046/j.1439-0469.2002.00200.x> (2002).

13 Struck, T. H., Purschke, G. & Halanych, K. M. A scaleless scale worm: Molecular evidence for the phylogenetic placement of Pisione remota (Pisionidae, Annelida) Published in collaboration with the University of Bergen and the Institute of Marine Research, Norway, and the Marine Biological Laboratory, University of Copenhagen, Denmark. *Mar. Biol. Res.* **1**, 243-253, <http://doi.org/10.1080/17451000500261951> (2005).
